# HIV-1 drug resistance and genetic transmission networks among patients with sexually transmitted HIV in Ningxia, China

**DOI:** 10.3389/fpubh.2024.1485516

**Published:** 2025-01-15

**Authors:** Jianxin Pei, Zhonglan Wu, Bingqian Si, Chunhua Ma, Yichang Liu, Xiaofa Ma, Wenhe Kuai, Yinhao Zhang, Yong Li

**Affiliations:** ^1^Ningxia Center for Disease Control and Prevention, Yinchuan, Ningxia, China; ^2^College of Life Sciences, University of Ningxia, Yinchuan, Yinchuan, Ningxia, China; ^3^College of Public Health, Ningxia Medical University, Yinchuan, Ningxia, China

**Keywords:** China, HIV - human immunodeficiency virus, art, sexually transmitted, genetic transmission networks

## Abstract

**Background:**

Over the past decade, sexual transmission has become a dominant source of new HIV-1 infection in China. However, very few studies have been conducted to characterize the two sexual transmissions, homosexual and heterosexual transmission. This study was conducted to better understand the relationship between genotypes, drug resistance, and molecular transmission networks in two groups of sexually transmitted HIV-1 in Ningxia, China.

**Methods:**

Plasma samples were collected from sexually transmitted HIV/AIDS patients in Ningxia between 2020 and 2021 for RNA extraction followed by HIV-1 genome sequencing, genotype and drug resistance analyses. The TN93 model in HyPhy2.2.4 with 1.25% as the threshold, was used to calculate the gene distance, and Cytoscape3.7.0 was used to generate a visual molecular transmission network.

**Results:**

A total of 269 samples were successfully sequenced, and 10 HIV-1 subtypes were detected. The two most common subtypes were CRF07_BC and CRF01_AE. All 10 subtypes were detected in heterosexually transmitted patients, and 7 subtypes were found in homosexually transmitted patients who were exclusively men sex with men (MSM). The drug resistance rates of heterosexual individuals and MSMs were 45.34 and 33.33%, respectively. Sequences from 120 patients entered the molecular transmission network, forming 35 clusters. The clustering rate for MSM (52.78%) was higher than that of heterosexual individuals (39.13%). Some MSM and HSTs were involved in the same cluster and might act as bridges for transmission between the two populations.

**Conclusion:**

Our data showed that heterosexually transmitted HIV-1 was more likely to be a drug-resistant virus, whereas MSM was more likely to contract viruses through network connection. It is strongly recommended that resistance testing be conducted before ART to improve effective treatment and reduce the spread of resistant viruses. Molecular networks can help to identify transmission clusters and provide more precise interventions.

## Background

Since the first case of AIDS was reported in the United States in 1981, human immunodeficiency virus type 1 (HIV-1) has spread worldwide through blood, mother-to-child vertical transmission, and sexual contact routes, posing a considerable threat to the economic and social development of societies (1). According to UNAIDS statistics, by the end of 2021, approximately 38.4 million people worldwide will be living with HIV, including 1,148,000 people living with HIV in China, and approximately 650,000 people will have died from HIV-related diseases in 2021 (2), making it one of the most important infectious diseases affecting public health. HIV was formerly spread mostly through blood transfusions and intravenous drug use in China; however, in recent years, the prevalence of sexually transmitted infections has greatly increased and has been considered the primary method of HIV transmission (3). The percentage of heterosexual and homosexual transmission in China has increased from 48.3 and 9.1% in 2009 to 74.2 and 23.3% in 2020, respectively (4). Due to unprotected sexual activity, multiple sexual partners, participation in commercial sex, bisexual sex, and other factors (5), men who have sex with men (MSM) have recently been identified as a high-risk population for HIV transmission. A study reported that MSM in China have a 19-fold higher risk of contracting HIV than the general population (6). However, heterosexual activities, such as its large population base, can spread HIV to the broader population, which presents substantial obstacles for HIV/AIDS prevention and treatment (7).

The National Free ART Program (NFATP) has greatly boosted the coverage of HIV/AIDS patients receiving antiviral therapy in China since its implementation in 2003 (8). Early antiretroviral therapy (ART) not only greatly reduces viral load but also leads to a significant reduction in the prevalence of risky sexual behaviors in different populations (9), thus greatly enhancing the effectiveness of HIV transmission prevention (10). However, with the increasing coverage of ART, the prevalence of drug resistance is increasing, which can substantially lessen the benefit of ART and may cause virological failure due to the high variation in the HIV gene and the pressure of drug selection (11). The reported data showed that 39% of patients for whom ART failed carried drug-resistant HIV-1, and the acquired drug resistance prevalence was 44.7% (12). HIV infections that are resistant to one or more antiviral drugs can spread via various routes. However, sexually transmitted individuals usually have poor treatment compliance, which could encourage the emergence and spread of drug resistance (13).

The Ningxia Hui Autonomous Region (Ningxia) is located in northwestern China and has a mixture of Han (64.05%) and Hui (35.04%) populations. The HIV-1 epidemic has been well controlled, but the case fatality rate has not shown a substantial downward trend, suggesting that the treatment of HIV-1 infection needs to be modified (14). It is also noted that the infections of HIV-1 are sexually spreading from high-risk populations to the general population in the region, indicating that the control measures for sexual transmission may not be very optimistic (14). Sexually transmitted HIV usually occurs through networks of closely connected groups. Building a molecular transmission network based on the genetic similarities of gene sequences of HIV-positive individuals is a useful approach to identify the transmission pathway and direct precise action against high-risk sources of infection (15). In this study, we combined drug resistance analysis and molecular transmission network technology to identify risk factors for HIV sexual transmission.

## Methods

### Study participants

HIV-1/AIDS patients were recruited if they met the following criteria: (1) HIV positive screened by HIV-1/HIV-2 enzyme-linked immunosorbent assay and confirmed by HIV-1 Western blotting in the Ningxia Center for Disease Control and Prevention (CDC); (2) the route of infection was sexual transmission, including MSM and heterosexual transmission; (3) received antiviral therapy for more than 6 months; (4) the viral load was greater than 400 copies/mL; and (5) the individuals voluntarily participated in this study with verbal or written informed consent. Demographic characteristics (ethnicity, sex, age, and marital status) were obtained from the Ningxia Information System for Disease Control and Prevention. After eliminating duplicate samples, pol sequence (covering 1,060 base pairs, HXB2:2253–3,313) information was successfully exported from 269 people living with HIV (PLWH) in this study.

### Laboratory tests

Plasma samples were separated from whole blood and preserved in a freezer at −80°C. RNA was extracted from 200 μL of plasma using an automatic nucleic acid extractor (Zhuhai Lizhu Reagent Co. LTD), which was performed in strict accordance with the manufacturer’s instructions. The HIV-1 pol gene was amplified by nested polymerase chain reaction (nPCR) using an in-house method (16), including the full-length protease gene (codon 1–99) and the first 300 amino acids of the reverse transcriptase gene (codon 1–300). The amplified product was approximately 1.1 kb. The amplified products were subjected to electrophoresis on 1% agarose gel, and the positive products were purified and sequenced by Beijing Nuosai Gene Sequencing Ltd.

### Sequence analysis

Initially, Sequencher 4.9 software was used to edit and assemble the sequences, followed by sequence alignment and arrangement using BioEdit 7.1.3. A phylogenetic tree was constructed using Mega7.0, using neighbor joining (Kimura two-parameter model, bootstrap samples = 1,000), and the HIV-1 subtypes were compared to international reference strains from the HIV gene database (LANL).^1^ If the sequence in which the genotype could not be confirmed by the phylogenetic tree or HIV BLAST database was considered a unique recombinant form (URF), recombination breakpoint analysis was performed using the SimPlot software.

### Drug resistance analysis

The obtained nucleic acid sequence was submitted to the Stanford University Drug Resistance Gene Database ^2^for base-resistance mutation identification and drug sensitivity analysis (17). According to the score in the database system, the degree of drug resistance was divided into five levels: sensitive (S), potential low drug resistance (P), low drug resistance (L), moderate drug resistance (I), and high drug resistance (H). At least one low-to-high resistance mutation indicates drug resistance.

### Genetic transmission network analysis

Pairwise gene distance of the sequence was calculated using the TN93 model in HyPhy2.2.4. The total number of transmission clusters in the molecular transmission network was considered to reach a maximum when the gene distance was less than 0.0125 (18). Therefore, gene distance was selected to construct the molecular transmission network, and Cytoscape 3.7.2 software was used to visualize the network. In molecular transmission networks, the number of edges connected to each node is a “degree” value.

### Statistical analysis

The database was constructed using Excel 2010 software, and the differences in transmission routes under different characteristics were statistically analyzed using SPSS 22.0. The count data were described as the number of cases and constituent ratios, and the differences between groups were compared using the chi-square test or Fisher’s exact test. Logistic regression analysis was used to determine the factors affecting the molecular transmission networks. All tests were two-tailed, and a *p* value <0.05 was considered statistically significant.

## Results

### Participant characteristics

A total of 3,524 patients received antiviral treatment between 2020 and 2021 in Ningxai and 332 of them had a viral load greater than 400 copies/mL. The intact pol sequence was successfully obtained from 269 patients that were included in this analysis.

108 patients were homosexual transmitted who were exclusively MSMs, while 161 infected individuals were heterosexual transmitted. The major characteristics of two group patients included (Table 1): (1) Heterosexual transmission occurred mainly among males, accounting for 75.78%; (2) The age range of MSM was 15–63 years, with the mean ± SD of 37.21 ± 10.87 years; and the age range of HSTs was 19–81 years and the mean ± SD of the age was 46.19 ± 15.63 years. The means of age of the two groups were statistically different. (3) The majority of patients in both groups lived in Yinchuan City, the capital city of Ningxia. (4) Of the MSM, 55.56% were unmarried, while only 26.72% of HSTs were unmarried. The difference in marital status in two group was statistically significant. (5) Approximately 80% of patients received first-line treatment, mainly lamivudine+efavirenz+tenofovir disoproxil fumarate (3TC + EFV + TDF) in both groups. (6) There was a trend that MSM had higher CD4+ T-cell counts than HSTs, and the mean CD4+ count in the two groups was statistically different. (7) 87.73% of patients had >10,000 copies/ml of viral load, and there was no statistically different between the two groups.

**Table 1:** Basic information of MSM and HSTs from 2020–2021 in Ningxia.

Characteristic	MSM (%)	HSTs (%)	*X* ^2^	*p*
Sex			/	/
Male	108 (100.00)	122 (75.78)		
Female	–	39(24.22)		
Age Mean ± SD	37.21 ± 10.87	46.19 ± 15.63	16.92	0.00
<30	27 (25.00)	24 (14.91)		
30–49	67 (62.04)	81 (50.31)		
≥50	14 (12.96)	56 (34.78)		
City of residence			12.77	0.01
Yinchuan	56 (51.85)	76 (47.20)		
Shizuishan	12 (11.11)	27 (12.42)		
Wuzhong	29 (26.85)	24 (14.91)		
Guyuan	5 (4.62)	24 (14.91)		
Zhongwei	6 (5.56)	10 (6.21)		
Marital status			30.33	0.00
Unmarried	60 (55.56)	43 (26.71)		
Married	21 (19.44)	78 (48.45)		
Divorced or widowed	26 (24.07)	40 (24.84)		
Unknown	1 (0.96)	0 (0.00)		
Treatment regimen			6.52	0.35
3TC + EFV + TDF	81 (75.00)	109 (67.70)		
AZT + 3TC + NVP	1 (0.93)	1 (0.62)		
AZT + 3TC + EFV	0 (0.00)	6 (3.72)		
TDF + 3TC + NVP	6 (5.56)	11 (6.83)		
TDF/AZT + 3TC + LPV/r	10 (9.26)	22 (13.66)		
Self-pay drug	5 (4.63)	7 (4.35)		
Others	5 (4.63)	5 (3.11)		
CD4+ T-cell count Mean ± SD	462.60 ± 270.33	351.18 ± 246.55	7.80	0.02
<200	22 (20.37)	52 (32.30)		
200 ~ 500	42 (38.89)	67 (41.61)		
>500	44 (40.74)	42 (26.09)		
VL copies/mL			2.66	0.26
<1,000	14 (12.96)	19 (11.80)		
1,000–10,000	19 (17.59)	42 (26.09)		
>10,000	75 (69.44)	100 (62.11)		
Drug-resistant HIV			3.87	0.05
Yes	36 (33.33)	73 (45.34)		
No	72 (66.67)	88 (54.66)		
Subtypes			3.00	0.39
CRF07_BC	67 (62.04)	89 (55.28)		
CRF01_AE	28 (25.93)	40 (24.84)		
B	5 (4.63)	14 (8.70)		
Others	8 (7.41)	18 (11.18)		

### Genotype analysis

A total of 10 HIV-1 subtypes were detected, including CRF07_BC (156 cases (57.99%)) and CRF01_AE (68 cases (25.29%)), followed by B (19 cases (7.06%)), CRF08_BC (8 cases (2.97%)), C (4 cases (1.49%)), CRF55_ 01 B (4 cases (1.49%)), and CRF59_ 01 B (1 case (0.37%)). In addition, seven cases of URFs (2.60%) and one case of CRF79_0107 (0.37%) were detected in this study.

**Figure 1:**
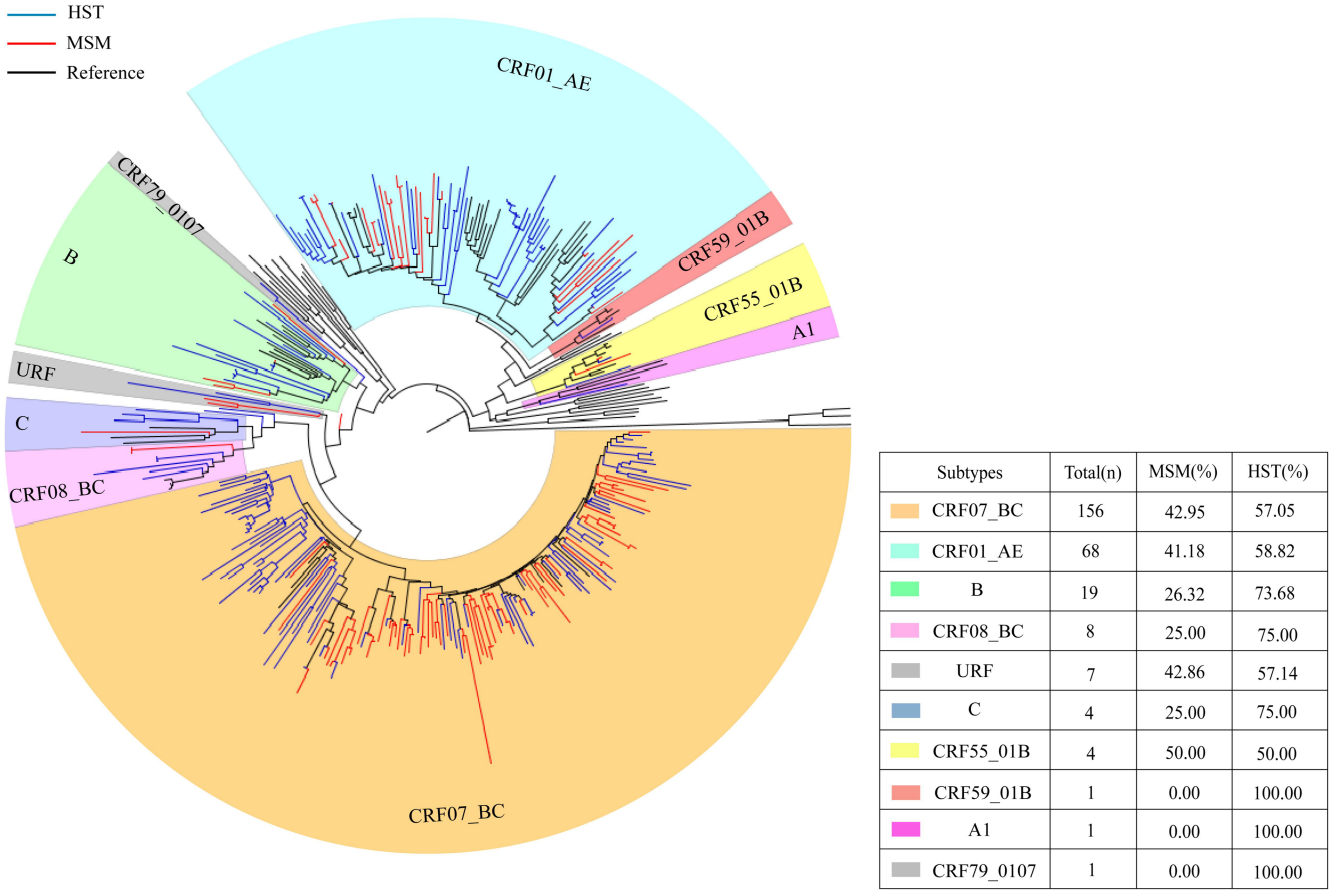
HIV-1 pol region phylogeny and gene subtype analysis of patients transmitted via MSM and HST. The blue branches indicate the sequences of HST, the red branches indicate the sequences from MSM, the black branches indicate reference sequences. A total of 156 sequences clustered with CRF07_BC, which HST making up 57.05% and MSM making up 42.95% (indicated by orange). 68 cases branched with CRF01_AE reference sequence, which HST accounted for 58.82% and MSM accounted for 41.18% (indicated by bluish green).19 cases of subtype B, with HST accounted for 73.68% and MSM accounted for 26.32% (indicated in grass green). 8 cases of CRF08_BC, which HST accounted for 75% and MSM accounted for 25% (indicated in light pink). 4 cases of subtype C, which HST accounted for 75% and MSM accounted for 25% (indicated in blue). 4 cases of CRF55_01B, with 50% of HST and MSM each (indicated in yellow).1 CRF59_01 B, only HST (indicated in red). 1 A1 detected in HST only (indicated in lavender).7 URF (HST 57.14%, MSM 42.86%) and 1 CRF 79 _ 0107 (HST detected only) (indicated in gray).

Different subtypes distributions were detected from patients of two different transmission routes. Seven subtypes were detected in the MSM group and 10 subtypes were detected in the HST group. CRF07_BC and CRF01_AE were the two major subtypes in both groups. The proportions of CRF07_BC and CRF01_AE subtypes in MSM were higher than those in HSTs, whereas subtypes CRF79_0107, CRF59_ 01 B, and A1 were only detected in HSTs (Figure 1).

### Drug resistance analysis

**Figure 2:**
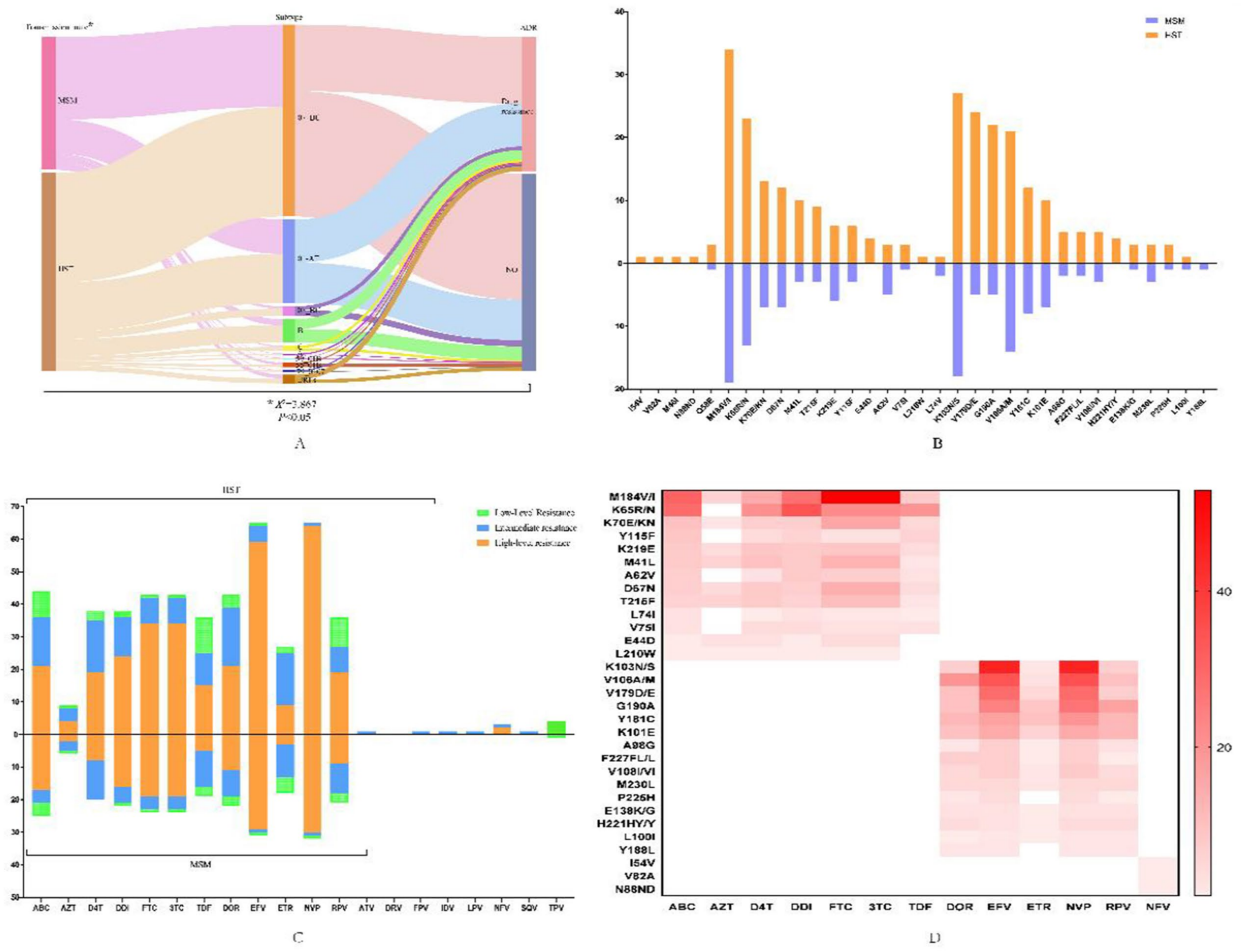
HIV-1 drug-resistant mutations and the profile of antiviral drug resistance in patients transmitted via MSM and HST. **(A)** Flow diagram of the relationship between infection routes, genotypes and drug resistance, which was analyzed by chi-square test. **(B)** Distribution of drug resistance sites in MSM and HST, in which the orange indicates HST and the blue indicates MSM. **(C)** Different antiviral drug resistance in MSM and HST, with the orange indicates high-level resistance, the blue indicates intermediate resistance and the green indicates low-level resistance. **(D)** Association of different antiviral drugs with major resistance sites that the darker color indicates greater effect of mutation sites on drugs. ABC, abacavir; AZT, zidovudine; D4T, stavudine; DDI, didanosine; FTC, emtricitabine; 3TC, lamivudine; TDF, tenofovir disoproxil fumarate; EFV, efavirenz; DOR, doravirine; NVP, nevirapine; RPV, Rilpivirine; ETR, etravirine; ATV/r, atazanavir/ritonavir; FPV/r, Fosamprenavir/ritonavir; IDV/r, indinavir/ritonavir; LPV/r, lopinavir/ritonavir, NFV, nelfinavir, SQV/r, saquinavir/ritonavir; TPV/r, tipranavir/ritonavir.

Among the 269 patients with successfully obtained sequences, HIV drug resistance mutations were detected in 109 individuals, and the resistance rate was 40.52% (109/269). The resistance rate in HST (45.34%, 73/161) was significantly higher than that in MSM (33.33%, 36/108), and there was a strong association between drug resistance and subtypes among the two groups (Figure 2A). Drug resistance to protease inhibitors (PIs), nucleoside reverse transcriptase inhibitors (NRTIs), and non-nucleoside reverse transcriptase inhibitors (NNRTIs) was found, and dual resistance to NRTIs/NNRTIs was dominant. The most common drug-resistant mutations in both MSMs and HSTs were NNRTI drugs, followed by NRTI drugs (Figures 2B,D). The sites and degrees of drug resistance were similar between the two groups (Figure 2C). There were four cases of PI-resistant mutations, and the most frequent mutation was Q58E, which was detected in both groups. The other PI-resistant mutations included I54V, V82A, M46I, and N88ND, which were detected only in HSTs (Figure 2B). All PI-resistant mutations showed low or intermediate resistance (Figure 2C). The main NRTI resistance mutation sites were M184V/I, K65R/KR/N, and K70E/R, and most were high resistance to 3TC, FTC, and abacavir (ABC). The main mutations responsible for NNRTI resistance were K103N/KN, V106M/A/I, and V179D/E/T, which were frequently detected in the CRF07_BC and CRF01_AE subtypes, and had the highest degree of resistance to NVP and EFV (Figures 2B–D).

### Genetic transmission network analysis

**Figure 3:**
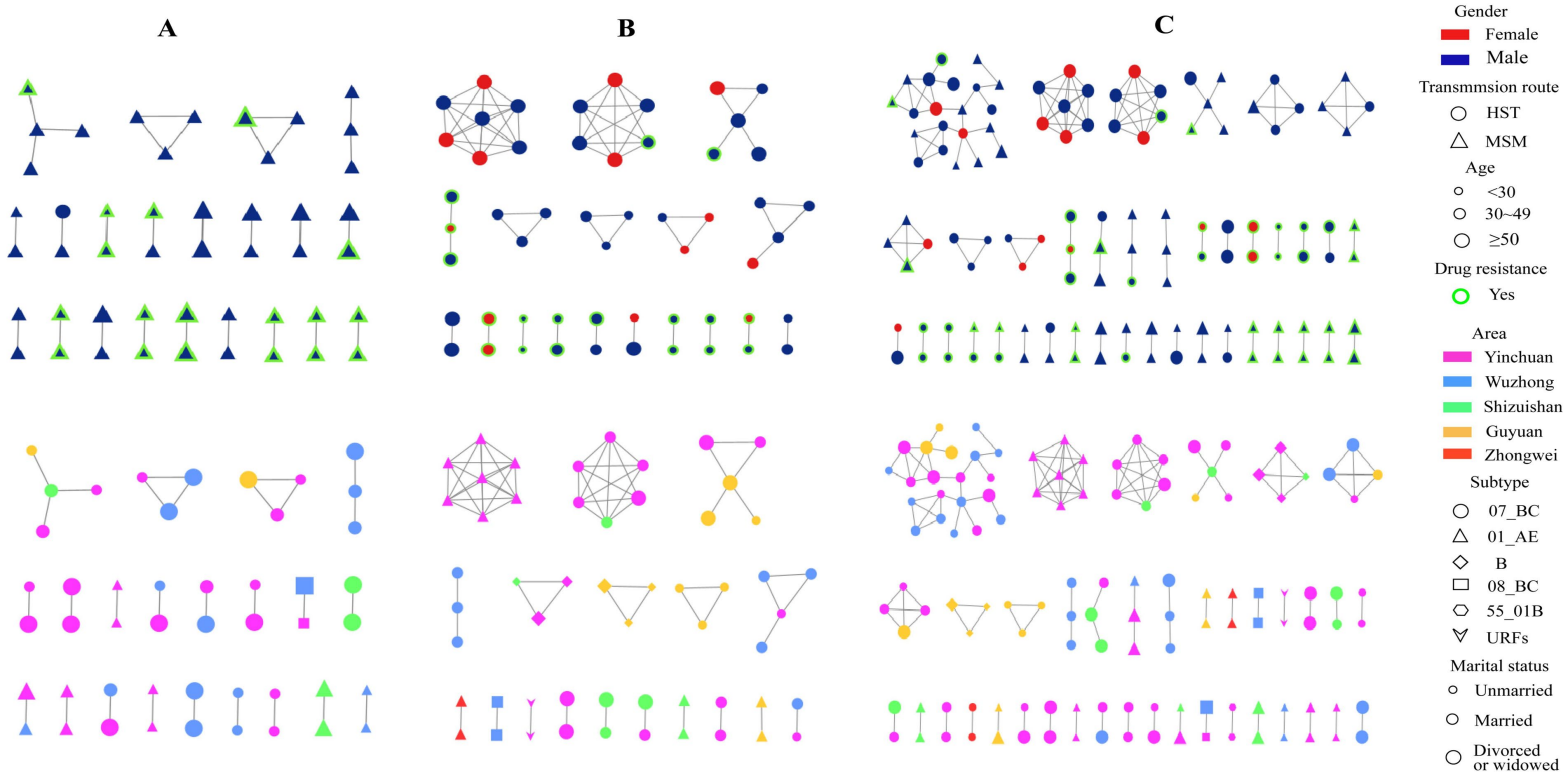
HIV-1 molecular transmission networks diagram. Top half: Molecular transmission network by gender, transmission route, age, and drug resistance, bottom half: Network by region, subtype, and marital status in MSM **(A)**, HST **(B)** and combined MSM and HST populations **(C)**.

All 269 sequences were selected to construct a molecular network, and the total number of transmission clusters in the network peaked at 39.120 sequences entering the network, with a network entry rate of 44.61% under a gene distance threshold of 1.25%. Among the 120 sequences entered the network, 57 sequences were from MSM and 63 sequences were from HSTs. The clustering rate of MSM (52.78%) was higher than that of HST (39.13%). Among the most common HIV subtypes, the cluster rates of subtypes CRF07_BC, CRF01_AE, and CRF08_BC were 48.72, 42.65 and 50%, respectively. This difference was not statistically significant (Figure 3C). By taking a variable factor entered the HIV molecular transmission network as a dependent variable, logistic regression analysis (Table 2) showed that heterosexual transmission and drug resistance increased the risk of HIV infection entering the transmission network (adjusted odds ratio (AOR) > 1, *p* < 0. 05). Married and divorced or widowed patients, those taking lopinavir/ritonavir (LPV/r), non-drug resistance mutation, and those with VL > 10,000 copies/ml were the factors that were less likely to be included in the transmission network (AOR < 1, *p* < 0. 05).

**Table 2:** Factors influencing HIV-1 molecular transmission networks via MSM and HSTs in 2020–2021.

	Total (%)	In cluster (%)	Not in cluster (%)	OR (95% CI)	*p*	AOR (95% CI)	*p*
Sex							
Male	230 (85.5)	105 (45.65)	125 (54.35)	1			
Female	39 (14.5)	15 (38.46)	24 (61.54)	1.362 (0.571–3.247)	0.486		
Transmission route							
MSM	108 (40.15)	57 (52.78)	51 (47.22)	1		1	
HSTs	161 (59.85)	63 (39.13)	98 (60.87)	2.847 (1.438–5.637)	0.003	2.571 (1.42–4.653)	0.002
Age							
<30	51 (18.96)	17 (33.33)	34 (66.67)	1			
30–49	148 (55.02)	65 (43.92)	83 (56.08)	0.796 (0.346–1.834)	0.593		
≥50	70 (26.02)	38 (54.29)	32 (45.71)	0.486 (0.179–1.32)	0.157		
City of residence							
Yinchuan	132 (49.07)	60 (45.45)	72 (54.55)	1			
Shizuishan	39 (14.5)	13 (33.33)	26 (66.67)	1.757 (0.743–4.156)	0.199		
Wuzhong	53 (19.7)	27 (50.94)	26 (49.06)	1.328 (0.608–2.9)	0.476		
Guyuan	29 (10.78)	16 (55.17)	13 (44.83)	0.54 (0.202–1.44)	0.218		
Zhongwei	16 (5.95)	4 (25)	12 (75)	3.376 (0.891–12.783)	0.073		
Marital status							
Unmarried	103 (38.29)	37 (35.92)	66 (64.08)	1		1	
Married	99 (36.8)	49 (49.49)	50 (50.51)	0.342 (0.154–0.759)	0.008	0.304 (0.154–0.599)	0.001
Divorced or widowed	66 (24.54)	34 (51.52)	32 (48.48)	0.404 (0.178–0.915)	0.03	0.333 (0.163–0.679)	0.002
Unknown	1 (0.37)	0 (0)	1 (100)	–	1	–	1
Treatment regimen							
3TC + EFV + TDF	190 (70.63)	82 (43.16)	108 (56.84)	1		1	
AZT + 3TC + NVP	2 (0.74)	1 (50)	1 (50)	0.76 (0.028–20.927)	0.871	0.885 (0.047–16.609)	0.935
AZT + 3TC + EFV	6 (2.23)	2 (33.33)	4 (66.67)	0.543 (0.077–3.802)	0.538	0.75 (0.12–4.689)	0.759
TDF + 3TC + NVP	17 (6.32)	10 (58.82)	7 (41.18)	0.452 (0.138–1.485)	0.191	0.404 (0.135–1.204)	0.104
TDF/AZT + 3TC + LPV/r	32 (11.9)	17 (53.13)	15 (46.88)	0.348 (0.142–0.852)	0.021	0.39 (0.168–0.908)	0.029
Self-pay drug	12 (4.46)	6 (50)	6 (50)	0.528 (0.127–2.194)	0.379	0.611 (0.17–2.192)	0.45
Others	10 (3.72)	2 (20)	8 (80)	3.806 (0.652–22.236)	0.138	3.752 (0.701–20.082)	0.122
CD4+ T-cell count							
<200	74 (27.51)	34 (45.95)	40 (54.05)	1			
200 ~ 500	109 (40.52)	45 (41.28)	64 (58.72)	1.383 (0.68–2.815)	0.371		
>500	86 (31.97)	41 (47.67)	45 (52.33)	1.031 (0.466–2.284)	0.939		
VL (copies/ml)							
1,000–10,000	61 (22.68)	20 (32.79)	41 (67.21)	1		1	
<1,000	33 (12.27)	14 (42.42)	19 (57.58)	0.723 (0.263–1.992)	0.531	0.721 (0.282–1.848)	0.496
>10,000	175 (65.06)	86 (49.14)	89 (50.86)	0.394 (0.191–0.812)	0.012	0.427 (0.222–0.822)	0.011
Drug-resistant HIV							
Yes	109 (40.52)	42 (38.53)	67 (61.47)	1		1	
No	160 (59.48)	78 (48.75)	82 (51.25)	0.429 (0.224–0.823)	0.011	0.453 (0.25–0.822)	0.009
Subtypes							
CRF07_BC	156 (57.99)	76 (48.72)	80 (51.28)	1			
CRF01_AE	68 (25.28)	29 (42.65)	39 (57.35)	0.999 (0.504–1.979)	0.997		
B	19 (7.06)	7 (36.84)	12 (63.16)	1.054 (0.326–3.406)	0.93		
CRF08_BC	8 (2.97)	4 (50)	4 (50)	0.9 (0.184–4.4)	0.896		
Others	18 (6.69)	4 (22.22)	14 (77.78)	2.409 (0.687–8.442)	0.17		

When only the MSM population was selected to construct a molecular network, a total of 21 transmission clusters (47 cases) were found, with a clustering rate of 43.52% (47/108) under the 1.25% gene distance threshold, in which only four were spread clusters with more than three nodes. All CRF 08_BC subtypes in the MSM population were included in the network, followed by a higher proportion of CRF07_BC and CRF01_AE. None of the other subtypes were included in the network (Figure 3A). When only HSTs were selected to construct the transmission network, a total of 18 transmission clusters (53 cases) were found, with a clustering rate of 32.92% (53/161) under the 1.25% threshold, in which eight transmission clusters had more than three nodes. The highest clustering rate of subtypes in the HSTs was 50% for URFs, followed by subtype B (42.86%), CRF07_BC (34.83%), CRF08_BC (33.33%), and CRF01_AE (30%) (Figure 3B).

## Discussion

Since China adopted Free ART Program in 2003 and ‘Treat for all’ policy in 2016, free of charge virus load test were implemented for all patients of the PLWHA receiving ART once per year. By analyzing the characteristics of HIV subtypes, drug resistance, and molecular transmission networks of homosexually and heterosexually transmitted infections in Ningxia, this study explored the general characteristics and risk factors of sexual transmission and provided useful information for improving control measures in different populations.

The results of this study confirmed the trends and main characteristics of HIV-1 infection that have been reported previously in Ningxia, which include the following: (1) men aged 30–49 were a high-risk population; (2) heterosexual transmission was the major sexual transmission route; (3) the majority of patients (79.93%) were taking first-line antiviral drugs; and (4) the primary gene subtypes were CRF07_BC and CRF01_AE. Compared with transmission among heterosexual individuals, MSM are younger, unmarried, and better-educated (19). However, MSMs are more likely to conceal their sexual orientation, which can cause intrafamily transmission and spread HIV to the general population (20).

There are reports showing different distribution of HIV-1 subtypes among different populations and regions (21, 22). Although a total of 10 HIV-1 subtypes were detected in the subjects of this study, CRF07_BC and CRF01_AE were the two dominant subtypes, which is consistent with the findings in other regions of China (23, 24). These two subtypes are the most prevalent strains of sexually transmitted HIV-1 in most regions. Subtype B is another major viral strain that spreads through sexual transmission, but it is usually overshadowed by the increasing prevalence of CRF01_AE (25). HIV-1 subtypes in Ningxia have recently become more diverse, with the feature of increasing detections of URFs. The study also found that the subtypes of heterosexually transmitted HIV were more diverse than those in MSM because subtypes A1, CRF79_0107, and CRF59_ 01 B were detected only in heterosexual individuals.

With increasing ART coverage, drug resistance mutations accumulate gradually, which may lead to treatment failure and further spread of HIV resistance (26). Some studies have shown that the incidence of drug resistance among MSM is higher than that among the general population (27). In this study, the drug resistance rate of individuals with sexually transmitted HIV was 40.52%, which was lower than the overall acquired drug resistance rate of 51.33%, which was calculated by a meta-analysis of results reported in China (28), indicating a regional difference in HIV-1 prevalence. The most common drug resistance in this study was NNRTIs, followed by NRTIs and PIs, which is consistent with previous reports (29). Among the drug resistance mutations in NRTIs, M184V/I, K65R/KR/N, and K70E/R were the main drug resistance sites. The M184V/I mutations have high and medium levels of resistance to 3TC and FTC, respectively, and the K65R mutation can reduce sensitivity to TDF (30). Among the drug resistant NNRTIs, K103N/KN, V106M/A/I, and V179D/E/T were the main mutations. Among them, the K103N caused a high level of resistance to the drugs NVP and EFV in patients receiving ART (31, 32).

In this study, under a gene threshold of 1.25%, the total clustering rate of sexual transmission in Ningxia was 44.61%. Heterosexual transmission and drug resistance increased the risk of HIV entering the transmission network (AOR > 1, *p* < 0.05), indicating that these two factors increased the risk of HIV transmission by the network (33, 34). These results are similar to those of a study conducted in the Eastern China Province, Jiangsu (35), Married, divorced, or widowed patients were less likely to be included in the transmission network. In other words, unmarried people usually have more social connections and are more likely to get HIV infection through their networks. The study also found that the patients who took LPV/r-containing medications and had a VL >10,000/mm^3^ were less likely to be included in the transmission network, which can be explained by the fact that LPV/r-containing medication had a lower chance of drug resistance (Figure 2) and high VL may limit individual social activities. The clustering rate of CRF08_BC was higher than that of CRF07_BC and CRF01_AE in our study, and all MSMs infected with the CRF08_BC subtype were included in the cluster. CRF08_BC has been reported to spread among different high-risk groups (injecting drug users and heterosexual individuals) to men who have sex with men (36, 37). Therefore, real-time monitoring of CRF08_BC subtype is necessary to prevent HIV-1 transmission (38).

Our analysis also found that MSMs and HSTs were involved in the same cluster (Figure 3C), indicating that some individuals acted as bridges for transmission between the two populations. Identification and control of these “bridge individuals, “who may have a bi-sexual orientation, are very important to curb the spread of HIV in different populations.

### Limitation of this study

This study had some limitations. First, patients with sexually transmitted HIV who received ART were selected and other routes of transmission were not included. As a result, the findings may not be generalizable to the entire population. Selection of patients with high viral loads and unsuccessful extraction of sequences from some samples can also generate bias. Follow-up studies should consider including all HIV/AIDS patients to better explain and analyze the characteristics of HIV transmission and improve sample processing and sequencing. This was a cross-sectional study and active transmission clusters could not be identified. A real-time dynamic network analysis will be more meaningful to trace the transmission source and improve the credibility of the molecular network.

## Conclusion

Sexual transmission is the predominant source of new HIV-1 infection in Ningxia, and both heterosexual and homosexual (exclusively MSMs) transmissions play roles. Our study showed that heterosexual transmission has more diverse genotypes and a higher risk of drug resistance than homosexual transmission. The molecular transmission network analysis found an increased cluster rate when combined the sequences of MSMs and HSTs than the analysis of separately, indicating the transmission of HIV-1 between the two populations, to which should pay extra attention. To achieve more effective and precise HIV-1 prevention and patient care, it is important to strengthen genetic monitoring and real-time network analyses.

## Data Availability

The raw data supporting the conclusions of this article will be made available by the authors, without undue reservation.

## Footnotes

^1^ https://www.hiv.lanl.gov/

^2^ http://hivdb.Stanford.edu
