# Supplementary material for: HIV-1 drug resistance and genetic transmission networks among patients with sexually transmitted HIV in Ningxia, China
Source: Front Public Health. 2025 Jan 15;12:1485516. doi: 10.3389/fpubh.2024.1485516 (PMC11775904; doi:10.3389/fpubh.2024.1485516)
Supplement: Supplementary file 1 [file Data_Sheet_1.ZIP › Figures-3.pdf]

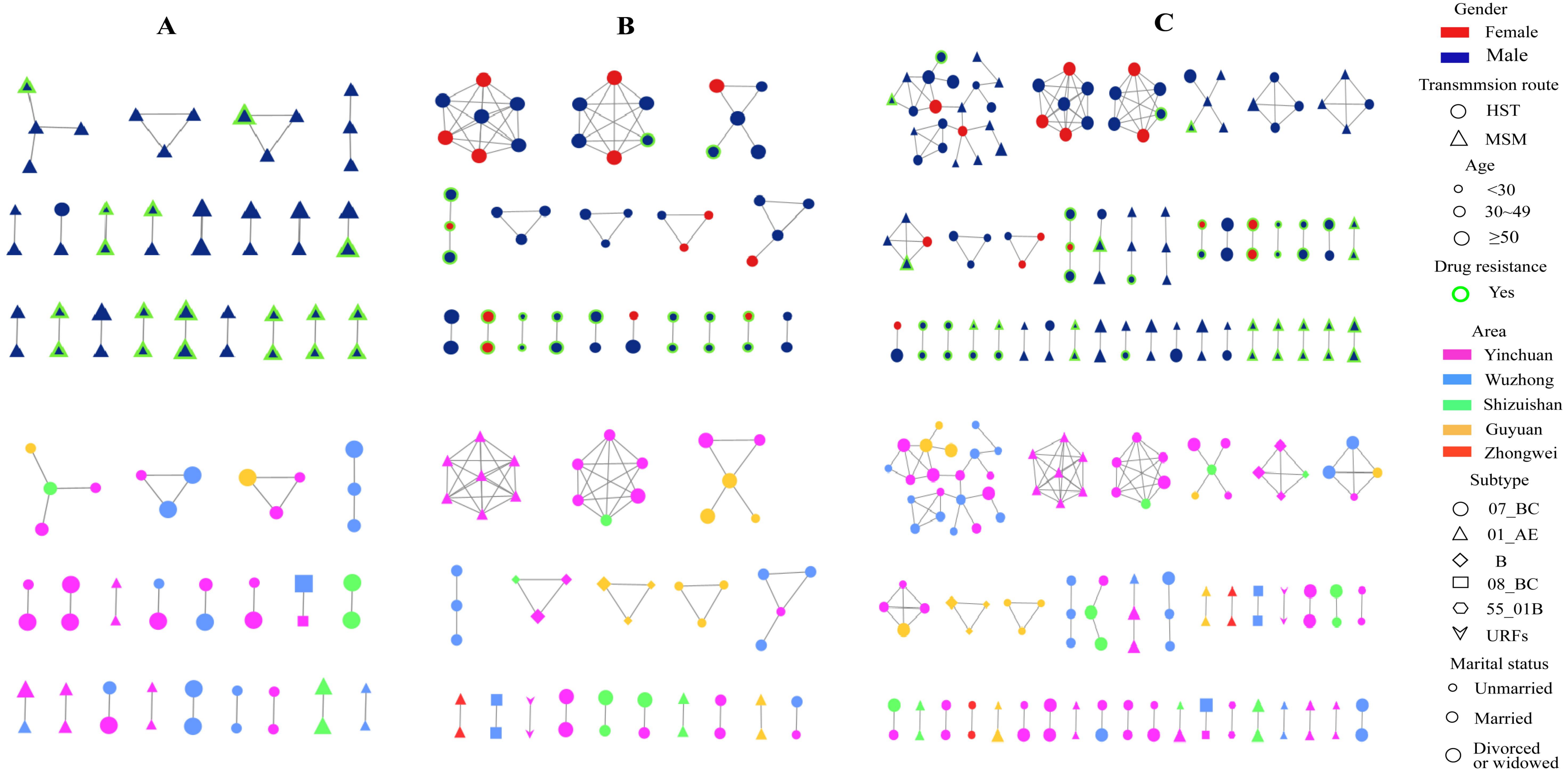

Figure 3 HIV-1 molecular transmission networks diagram. Top half: Molecular transmission network by gender, transmission route, age, and drug resistance, bottom half: Network by region, subtype, and marital status in MSM (A), HST(B) and combined MSM and HST populations (C).
