# Supplementary material for: HIV-1 drug resistance and genetic transmission networks among patients with sexually transmitted HIV in Ningxia, China
Source: Front Public Health. 2025 Jan 15;12:1485516. doi: 10.3389/fpubh.2024.1485516 (PMC11775904; doi:10.3389/fpubh.2024.1485516)
Supplement: Supplementary file 1 [file Data_Sheet_1.ZIP › Figures-1.pdf]

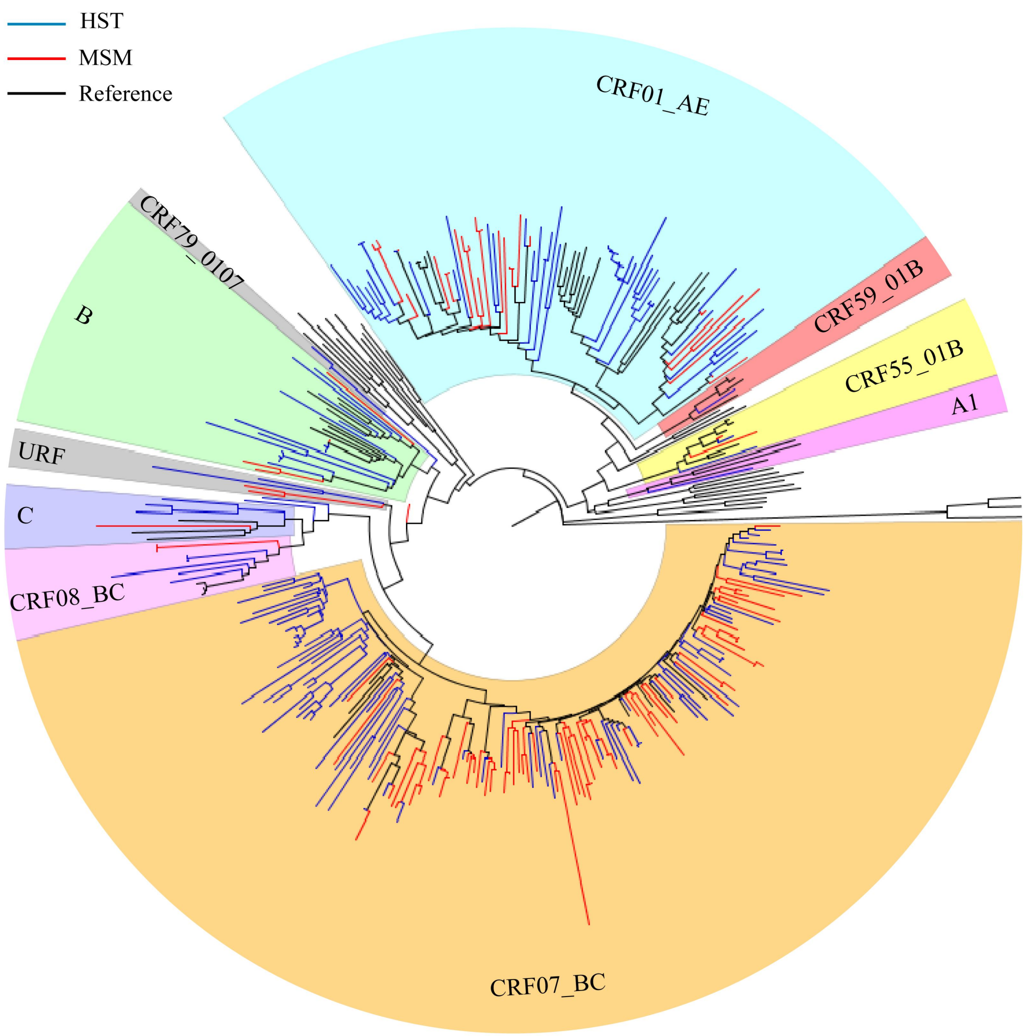

Subtypes	Total(n)	MSM(%)	HST(%)
CRF07_BC	156	42.95	57.05
CRF01_AE	68	41.18	58.82
B	19	26.32	73.68
CRF08_BC	8	25.00	75.00
URF	7	42.86	57.14
C	4	25.00	75.00
CRF55_01B	4	50.00	50.00
CRF59_01B	1	0.00	100.00
A1	1	0.00	100.00
CRF79_0107	1	0.00	100.00

Figure 1. HIV-1 pol region phylogeny and gene subtype analysis of patients transmitted via MSM and HST. The blue branches indicate the sequences of HST, the red branches indicate the sequences from MSM, the black branches indicate reference sequences. A total of 156 sequences clustered with CRF07_BC, which HST making up 57.05% and MSM making up 42.95% (indicated by orange). 68 cases branched with CRF01_AE reference sequence, which HST accounted for 58.82% and MSM accounted for 41.18% (indicated by bluish green).19 cases of subtype B, with HST accounted for 73.68% and MSM accounted for 26.32% (indicated in grass green). 8 cases of CRF08_BC,which HST accounted for 75% and MSM accounted for 25% (indicated in light pink). 4 cases of subtype C, which HST accounted for 75% and MSM accounted for 25% (indicated in blue). 4 cases of CRF55_01B, with 50% of HST and MSM each (indicated in yellow).1 CRF59_01 B, only HST (indicated in red). 1 A1 detected in HST only (indicated in lavender).7 URF (HST 57.14%, MSM 42.86%) and 1 CRF 79 _ 0107 (HST detected only) (indicated in gray).
