# Supplementary material for: HIV-1 drug resistance and genetic transmission networks among patients with sexually transmitted HIV in Ningxia, China
Source: Front Public Health. 2025 Jan 15;12:1485516. doi: 10.3389/fpubh.2024.1485516 (PMC11775904; doi:10.3389/fpubh.2024.1485516)
Supplement: Supplementary file 1 [file Figures-2.pdf › Figures-2.pdf]

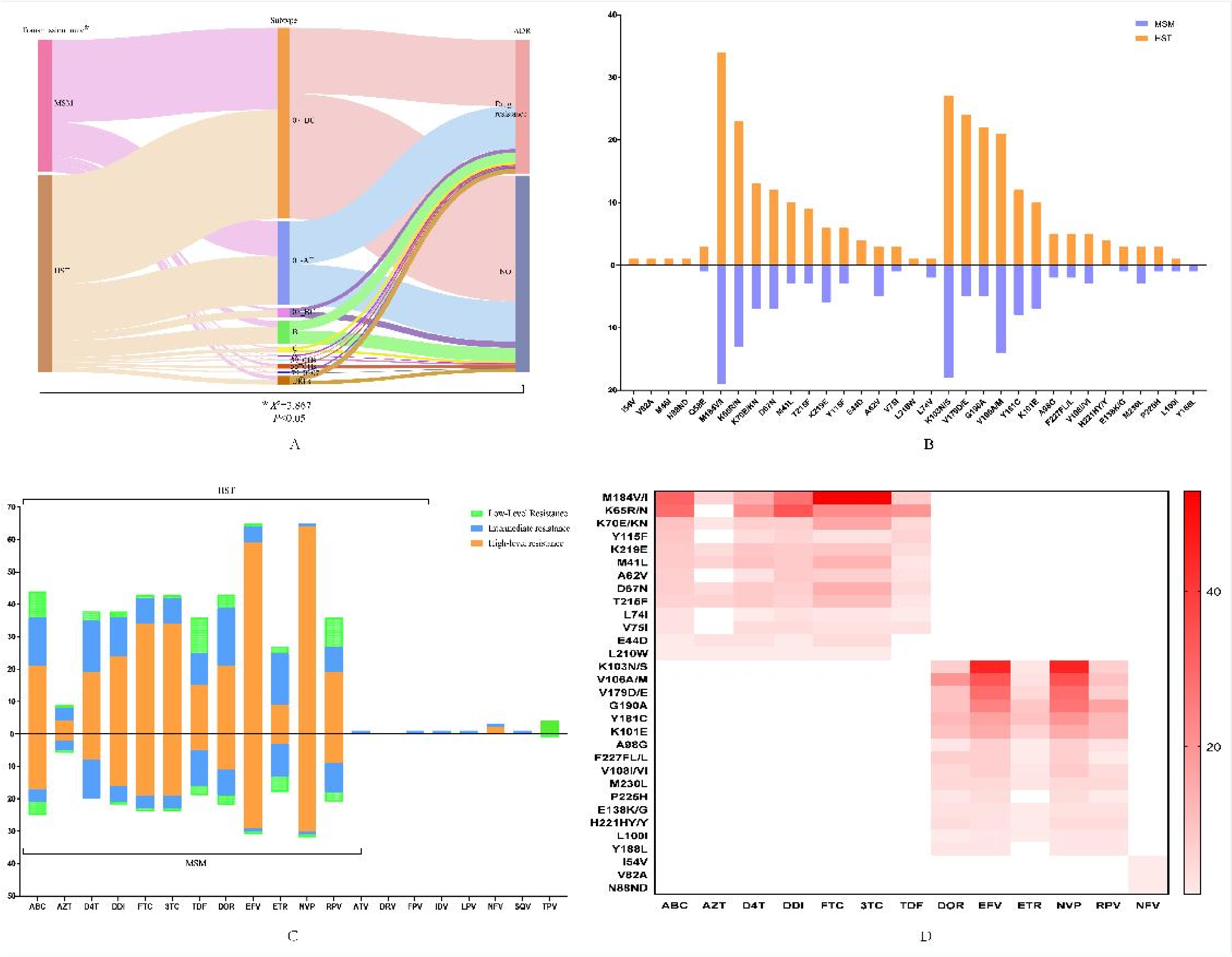

Figure 2 HIV-1 drug-resistant mutations and the profile of antiviral drug resistance in patients transmitted via MSM and HST. A. Flow diagram of the relationship between infection routes, genotypes and drug resistance, which was analyzed by chi-square test. B. Distribution of drug resistance sites in MSM and HST, in which the orange indicates HST and the blue indicates MSM. C. Different antiviral drug resistance in MSM and HST, with the orange indicates high-level resistance, the blue indicates intermediate resistance and the green indicates low-level resistance. D. Association of different antiviral drugs with major resistance sites that the darker color indicates greater effect of mutation sites on drugs. ABC: abacavir; AZT: zidovudine; D4T: stavudine; DDI: didanosine; FTC: emtricitabine; 3TC: lamivudine; TDF: tenofovir disoproxil fumarate; EFV: efavirenz; DOR: doravirine; NVP: nevirapine; RPV: Rilpivirine; ETR: etravirine; ATV/r: atazanavir/ritonavir; FPV/r: Fosamprenavir/ritonavir; IDV/r: indinavir/ritonavir; LPV/r: lopinavir/ritonavir, NFV: nelfinavir, SQV/r: saquinavir/ritonavir; TPV/r: tipranavir/ritonavir.
